# Reconstruction of Positron Emission Tomography Images Using Gaussian Curvature

**DOI:** 10.1155/2018/4706165

**Published:** 2018-11-15

**Authors:** Jose Mejia, Boris Mederos, Jie Zhao, Leticia Ortega, Nelly Gordillo

**Affiliations:** ^1^Department of Electrical and Computation Engineering, Universidad Autonoma de Ciudad Juarez, Ciudad Juarez 32310, Mexico; ^2^Department of Physics and Mathematics, Universidad Autonoma de Ciudad Juarez, Ciudad Juarez 32310, Mexico; ^3^School of Medical Imaging, Xuzhou Medical University, XuZhou, China

## Abstract

Positron emission tomography (PET) provides images of metabolic activity in the body, and it is used in the research, monitoring, and diagnosis of several diseases. However, the raw data produced by the scanner are severely corrupted by noise, causing a degraded quality in the reconstructed images. In this paper, we proposed a reconstruction algorithm to improve the image reconstruction process, addressing the problem from a variational geometric perspective. We proposed using the weighted Gaussian curvature (WGC) as a regularization term to better deal with noise and preserve the original geometry of the image, such as the lesion structure. In other contexts, the WGC term has been found to have excellent capabilities for preserving borders and structures of low gradient magnitude, such as ramp-like structures; at the same time, it effectively removes noise in the image. We presented several experiments aimed at evaluating contrast and lesion detectability in the reconstructed images. The results for simulated images and real data showed that our proposed algorithm effectively preserves lesions and removes noise.

## 1. Introduction

Positron emission tomography (PET) is a technique used for medical imaging, providing images that represent the evolution of various biochemical and physiological processes in different tissues. In addition, PET imaging complements other clinical imaging modalities, such as computed tomography (CT) and magnetic resonance (MR), providing additional metabolic data to support the anatomic information [1, 2].

PET is used extensively as a clinical tool in oncology for noninvasively monitoring and grading tumors, as well as determining tumor recurrence; it is also employed to observe the effects of therapeutic treatment. To acquire the image, the patient is injected with a radioactive substance, or a radiotracer, which is designed to target the body tissues of interest. The radiotracer emits positrons that subsequently produce a pair of gamma rays, which are detected by the PET system; finally, the image is produced via the counts of the emitted gamma rays. However, because the radiotracer's permitted doses are small, image reconstruction from low-count PET is difficult, which is considered an inverse ill-posedness problem; in addition to the low count issue, other factors, such as scattering, random events, and the sensors' death time, contribute to generating noisy images [3]. To improve the quality of the reconstructed image and account for the ill-posedness problem, a regularization term is incorporated.

Regularization in PET has been addressed in several works. In [1], it was suggested that the total variation (TV) could be included to preserve the edges and boundary surfaces; these researchers developed a GPU-based implementation. In [4], TV regularization was used on both the image and projection spaces, via a formulation of the variational problem that considered the TV penalty terms on both the image and sinogram, the researchers showed that their scheme performed better on reconstructing images with thin structures. In [5], a method combining expectation maximization (EM) and TV regularization, called EM + TV, was proposed. These researchers proved that the method can reconstruct better images using fewer views. The authors in [6] proposed nonlocal regularization; their regularizer was designed to selectively consider and use anatomical information from other sources, such as CT, only when the information was reliable. In this way, they made the regularization term more robust to the signal mismatch problem caused by the indirect relationship between the PET image and the anatomical images from other sources. In [7], an anisotropic diffusion term was introduced; in combination with a median filter, this was designed to preserve the edges effectively. The authors reported that this solution suppressed noise and preserved the structure of the image's edges.

Recently, geometry-driven image diffusion techniques have been proposed to solve and regularize ill-posedness problems; such techniques use the local geometric properties of the image surface, such as the curvature and mean curvature (MC). Gaussian curvature (GC) was proposed recently in [8]; it was used as a diffusion equation for noise removal, demonstrating advantages for edge and small feature preservation. Moreover, the authors in [9] proposed regularization combined with Bayesian reconstruction. They used an a priori method based on MC and GC, and the experimental results showed that curvature regularizers are suitable for reducing noise while preserving edges.

Given the good results obtained using curvature-based terms, we propose the use of weighted Gaussian curvature (WGC) applied to the problem of reconstructing PET data. WGC was proposed for denoising and cartoon/texture decomposition in [10]; we show that WGC performs better than GC does, while exhibiting rapid convergence and good adaptability.

The rest of the paper is organized as follows: in Section 2, the observational model for PET reconstruction is explained, the GC framework is revised, and the proposed algorithm is presented.  Section 3 details the results of the experiments. Finally, conclusions are provided in Section 4.

## 2. Materials and Methods

### 2.1. PET Geometry

**Figure 1:**
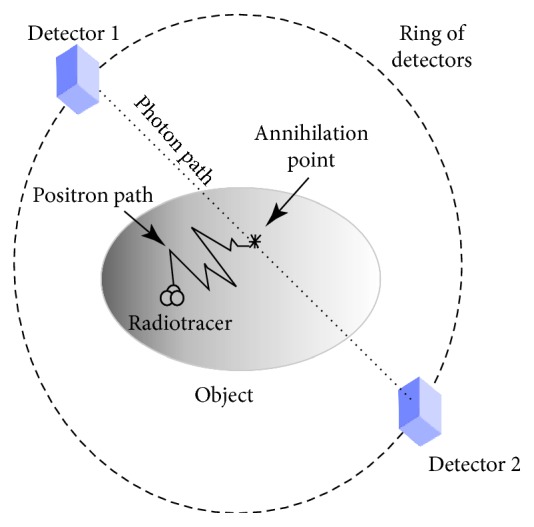
Ring of a positron emission tomography (PET) scanner. The object under study is injected with a radiotracer; this emits a positron that is eventually annihilated by an electron after traveling a short distance in the object (positron path). The annihilation produces two high-energy photons traveling in opposite directions, which are detected by the scanner sensors (detectors).

After injecting the patient with the radiotracer, the acquisition process for collecting the data for a PET scan begins with the radioactive substance emitting positrons. When an emitted positron encounters an electron, a pair of gamma rays is produced by the annihilation of the electron-positron. The gamma rays are sent out in opposite directions, and subsequently, they are sensed by the PET scanner (Figure 1). The counts of all these pairs at different angles or projections are used to build the PET image, which resembles the distribution of the radiotracer in the body.

**Figure 2:**
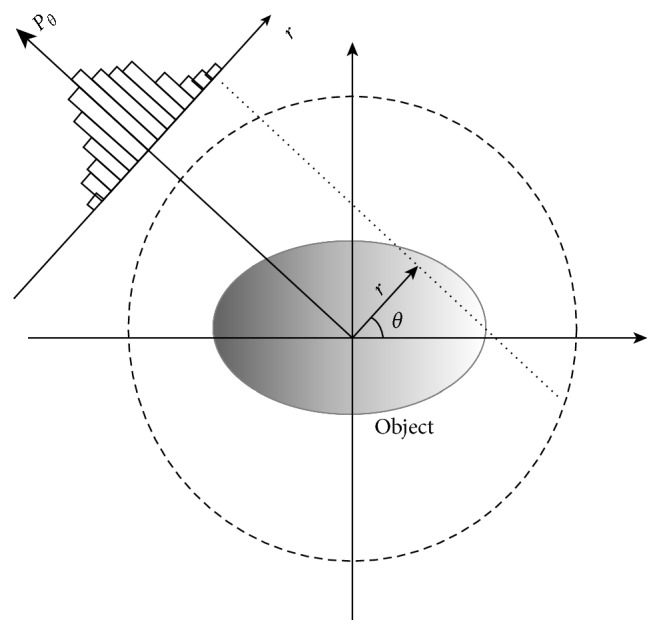
Projections. Events counted along the dotted line contribute to the histogram of the projection at angle *θ* and radius *r* from the center of the scanner; the histogram is completed by iterating along different *r* values. The process is repeated for each angle, and the total numbers of angles and projections are determined by the san geometry.

PET reconstruction algorithms aim at obtaining an image of the patient, given the radiotracer distribution in the body. For this purpose, the PET scanner has rings with sensor elements that detect and count the high-energy photons generated by the annihilation of positrons. The counts acquired at different angles form a projection, *P*_*θ*_ *(r)*, of the radiotracer's distribution. This is visualized as a histogram using the angle, *θ*, and distance, *r*, from the scanner center; then, each projection is stacked as a set of sinograms (Figure 2).

The sinogram is a common radon transform representation; in this case, it represents the radon transform of the object scanned. When a large amount of radiotracer is available, it is possible to accurately reconstruct the object from its projections by finding its inverse radon transform. However, because of the size of the scanner sensors and regulations on the radiotracer dose that can be administered to patients, among other things, the acquired data from the scanner usually contain a high level of noise and a limited number of counts. This makes direct radon transform infeasible; thus, alternative reconstruction methods are required.

The reconstruction problem can be posed as a system of linear equations as

(1) $$\begin{array}{c} Au=b, \end{array}$$

where *u* is a size, *N*=*N*_1_ *N*_2_ vector representing the object, *b* is the sinogram ordered as a vector of size *M*, and *A* is the so-called system matrix or radon transform matrix. Due to several factors, such as noise and a low event count, the inverse problem (1) is considered an ill-posedness problem; thus, it is necessary to regularize it to enforce stability [10].

An inverse problem to reconstruct the intensity of an object *u*, given a number of projections, *b*, can be formulated as

(2) $$\begin{array}{c} \underset{u}{\min}{\left\|Au-b\right\|}^{2}+\lambda \Phi \left(u\right), \end{array}$$

where Φ(·) is a suitable regularization term. This is added because the inverse problem is typically ill-posed, and thus, some type of regularization is needed to produce a reasonable reconstruction and emphasize a priori information about the characteristics of the class of image to be reconstructed. In addition, the parameter *λ* is a scalar regularization coefficient. It should be noted that Equation (2) does not consider that the noise on sinogram data *b* is Poisson distributed. This is because the counting process of the positron emissions occurs following a spatial Poisson point process [3].

In this paper, for the regularization of the ill-posedness problem, we consider a term Φ(·) corresponding to the WGC, as described in [10].

### 2.2. Gaussian Curvature

The Gaussian curvature is calculated as the product of the principal curvatures; thus, at points where any of the principal curvatures is zero, GC is also zero, in contrast to the MC, which averages the principal curvatures and is not necessarily zero at these points. This allows GC to better preserve structures than MC can; for example, the edges of the image generally have a large maximum principal curvature value across the edge, while the minimum principal curvature along the edge is nearly zero [8]. In addition, structures that are not preserved by gradient-based methods because of their low gradient magnitudes can be preserved under the GC scheme. This is true in the case of ramp-like structures, which generally have zero GC [8]. In addition, structures created by noise usually have high GC values, and thus, the noise is effectively removed.

The Gaussian curvature for a continuous surface, *u*, can also be determined from the ratio of the determinants of the second and first fundamental forms, defined as [10]

(3) $$\begin{array}{c} G\left(u\right)=\frac{u_{xx}u_{yy}-u_{xy}^{2}}{{\left(1+u_{x}^{2}+u_{y}^{2}\right)}^{2}}, \end{array}$$

where *x* and *y* are coordinates of a point in the image and *u*_*dc*_ is the second derivative with respect to *c* and *d*.

The GC method proposed in [8] includes a Gaussian curvature-dependent regularization term

(4) $$\begin{array}{c} \nabla \cdot \left(\phi \left(G\left(u\right)\right)\nabla u\right), \end{array}$$

where *ϕ*(·) is a nonnegative monotonically increasing function, ∇· is the divergence operator, and ∇ the gradient operator. In [6], the term (4) is used in a geometry-driven diffusion algorithm that takes into account the curvature of the surface represented by the image, and the curvature controls the amount of diffusion in each region. In [10], model with a weighted GC term, *W*(*u*)*G*(*u*), such a model is not based on anisotropic diffusion; in this work, we develop a method based on this scheme.

### 2.3. The Proposed Method

In this section, we provide the details of the proposed methodology. The variational techniques have already been used for reconstruction; examples of regularization terms include the TV and L1 norms [5, 7].

To adapt model (1) to the reconstruction problem to deal with Poisson noise, we formulate the problem using a discrete Bayesian framework and define the likelihood distribution, *P*(*b*∣|*u*), of the data, *b*, given the image, and *u*, as a Poisson distributed

(5) $$\begin{array}{c} P\left(\left.b\;|\;\right|u\right)=\overset{M}{\underset{i=1}{\prod }}\frac{e^{-{\left(Au\right)}_{i}}{\left({\left(Au\right)}_{i}\right)}^{b_{i}}}{b_{i}!}, \end{array}$$

where (·)_*i*_ refers to the *i*th element of the vector. For the a priori probability, in this paper, we explore the WGC, since it is known to be efficient for computation and generating satisfactory results when used in image smoothing for sharpening, denoising, and cartoon/texture decomposition [10]. Thus, we define the a priori probability as a Gibbs distribution:

(6) $$\begin{array}{c} P\left(u\right)=e^{-\lambda \Phi \left(u\right)}, \end{array}$$

where *λ* is a regularization parameter of the model and

(7) $$\begin{array}{c} \Phi \left(u\right)=\overset{N_{1}}{\underset{i=1}{\sum }}\overset{N_{2}}{\underset{j=1}{\sum }}W\left(u_{i,j}\right)\left(Gu_{i,j}\right), \end{array}$$

where *W*(*u*_*i*,*j*_) is a weight function defined as *W*(*u*_*i*,*j*_)≔(1+*d*_*x*_^2^+*d*_*y*_^2^)^2^, with *d*_*x*_ and *d*_*y*_ corresponding to the discrete first derivative of *u*_*ij*_ along the *j* and *i* components, respectively.

The image estimation is found through the posterior distribution

(8) $$\begin{array}{c} P\left(\left.u\;|\;\right|b\right)=\frac{P\left(\left.b\;|\;\right|u\right)p\left(u\right)}{P\left(b\right)}. \end{array}$$

To establish an estimate of the image $\hat{u}$, we use the maximum a posteriori (MAP) estimation procedure. We employ a*–*log function on *P*(*u*∣|*b*) to dispense with the exponentials and turn the problem into a minimization; thus, we obtain

(9) $$\begin{array}{c} \underset{u}{\arg\min}\overset{M}{\underset{i=1}{\sum }}\left({\left(Au\right)}_{i}-b_{i}\;\log{\left(Au\right)}_{i}\right)+\lambda \overset{N_{1}}{\underset{i=1}{\sum }}\overset{N_{2}}{\underset{j=1}{\sum }}W\left(u_{i,j}\right)G\left(u_{i,j}\right). \end{array}$$

For the weights, the components of the regularization term are given by

(10) $$\begin{array}{c} W\left(u_{i,j}\right)G\left(u_{i,j}\right)=\left({\left(u_{i,j}\right)}_{xx}{\left(u_{i,j}\right)}_{yy}-{\left(u_{i,j}\right)}_{xy}^{2}\right), \end{array}$$

where (*u*_*i*,*j*_)_*xx*_ and (*u*_*i*,*j*_)_*yy*_ correspond to the second discrete derivative of *u*_*ij*_ along the *j* and *i* components, respectively, and (*u*_*i*,*j*_)_*xy*_ correspond to the mixed derivative. The regularization term in (9) can then be expressed in a matrix form as follows [10]:

(11) $$\begin{array}{c} \overset{N_{1}}{\underset{i=1}{\sum }}\overset{N_{2}}{\underset{j=1}{\sum }}W\left(u_{i,j}\right)G\left(u_{i,j}\right)=u^{T}Du, \end{array}$$

where *D* is a matrix defined as

(12) $$\begin{array}{c} D=C_{xx}^{T}C_{yy}-C_{xy}^{T}C_{xy}, \end{array}$$

where *C*_*xx*_, *C*_*yy*_, and *C*_*xy*_ are matrices representing the central differences of the second derivatives of *u*_*i*,*j*_with respect to *i*, *j*, and in both directions. Consequently, the proposed corresponding functional can be stated as

(13) $$\begin{array}{c} J\left(u\right)=\overset{M}{\underset{i=1}{\sum }}\left({\left(Au\right)}_{i}-b_{i}\;\log\left({\left(Au\right)}_{i}\right)\right)+\frac{1}{2}\lambda u^{T}Du. \end{array}$$

The reconstructed image $\hat{u}$ is found as the minimum of

(14) $$\begin{array}{c} \hat{u}=\underset{u}{\arg\min}\overset{M}{\underset{i=1}{\sum }}\left({\left(Au\right)}_{i}-b_{i}\;\log\left({\left(Au\right)}_{i}\right)\right)+\frac{1}{2}\lambda u^{T}Du. \end{array}$$

Furthermore, the Euler–Lagrange equations are given by

(15) $$\begin{array}{c} J^{\prime }\left(u\right)=\lambda \left(D^{T}+D\right)u+\overset{M}{\underset{i=1}{\sum }}\left(a_{i}\left(1-\frac{b_{i}}{{\left(Au\right)}_{i}}\right)\right)=0. \end{array}$$

To solve the minimization problem, we use an iterative semi-implicit finite-difference scheme. By employing (14) and (15), we followed a simple iterative scheme using the evolution equation

(16) $$\begin{array}{c} \partial_{t}u=-J^{\prime }\left(u\right), \end{array}$$

which corresponds to the gradient descent of (13). The iterative scheme, with step size *τ*, used to solve the problem of reconstruction is presented in Algorithm 1.

For the experiments, we determined the *λ* and step size *τ* parameters using grid search evaluating the peak signal to noise ratio (PSNR); we choose the combination of values giving higher PSNR, *λ* = 2.5 and *τ* = 0.0005.

In this paper, the weighting scheme used was the same as [10], where weight function *W* is proportional to a small surface area element, and is mainly chosen to simplify the final solution. Here, we can relate to PET as an element of areas of surfaces of interest such as organs with activity concentrations. The *W* can also be chosen as the nonlocal mean weights for each pixel or as an edge indicator function in order to enforce the edges between regions of different levels of activity in the pet image while in homogenous areas reduce the noise.

### 2.4. Experiments

**Figure 3:**
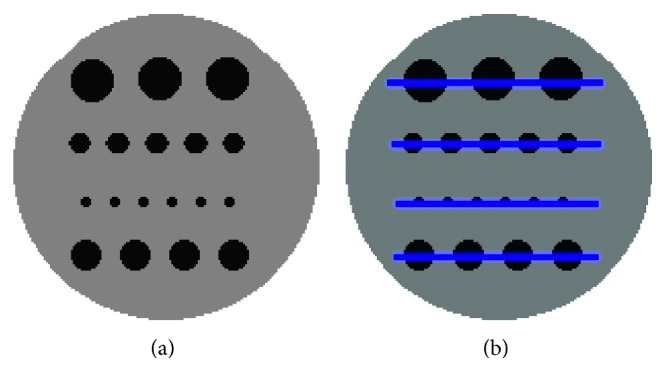
(a) Phantom holes consisting of four rows of cylinders of 2, 3, 4, and 5 mm. (b) Lines indicating where the profiles were taken.

We conducted several experiments to evaluate different aspects of the reconstruction method. The first experiment was designed for evaluating the resolution that can be achieved with the proposed method with reconstructed images from low-count data. To accomplish this, we used the phantom employed in [11], consisting of a 16 mm cylinder filled with water. Inside the cylinder are four rows of small cylinders of 2, 3, 4, and 5 mm in diameter; they are filled with activity equivalent to an ^18^F-fluorodeoxyglucose concentration of 1 : 8 in relation to the background.  Figure 3 shows the phantom. For the reconstruction of this phantom, a system matrix of size 16,768 × 8,100 was used. From the reconstructed images using the different methods, we obtained line profiles along each row and graphics showing the performance of each method to follow the profile of the original phantom. Also, measures of the PSNR and structural similarity index (SSIM) are provided.

**Figure 4:**
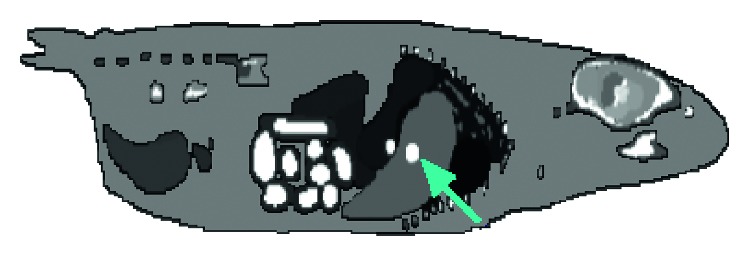
The MOBY phantom [12], with a lesion in the liver indicated by the arrow.

In a second experiment aimed at quantitatively evaluating the reconstruction quality under the context of lesion detectability, we used the MOBY phantom, which provides a detailed structure of the anatomy of a mouse, as described in [12]. Inside the phantom, we simulated a lesion in the lung by adding a small sphere of 1 mm in radius; we also simulated activity equivalent to an ^18^F-fluorodeoxyglucose radiotracer, with a relative lesion-to-background radioactivity ratio of 4 : 1.  Figure 4 shows a section of the MOBY phantom, with the lesion indicated by an arrow. To quantitatively evaluate lesion detectability, we used a channelized Hotelling observer [13], and as a figure of merit for this study, the area under the curve (AUC) was calculated.

Mathematical model observers are used as predictors of human lesion detection performance with PET images [14]. Specifically, the channelized Hotelling observer has become a widely used approach for the assessment of PET medical image quality, as it accurately models human observers and is used frequently for the early assessment and optimization of imaging devices and algorithms [15].

The task given to the model observer was to try to detect a lesion with a known location on a two set of images, one with and the other without lesions. This is known as the signal-known-exactly task.

The images presented to the observer were simulated from a set of 15 realizations of the MOBY mouse phantom with a lesion, as well as a set of 15 realizations without lesions. The two sets of images were processed with each method; following this, the results were fed to the observer. From the observer output, a receiver operating characteristic (ROC) curve was constructed, and its AUC was calculated. Our goal was to objectively quantify the improvement in lesion detectability after processing with each method.

PET scans of both phantoms—the cylinders and MOBY—were simulated using the Simulation System for Emission Tomography (SimSET) software [16]. SimSET uses Monte Carlo techniques for modeling the physical processes and instrumentation of a PET scanner.

In the final study, we evaluated the performance with the measured data. We used data from a subject who was scanned on a CTI ECAT PET scanner the raw sinograms, where acquired at 160 radial samples and 192 angular samples; data were precorrected for delayed coincidences. Data are available at http://web.eecs.umich.edu/∼fessler/ [17].

## 3. Results and Discussion

In this section, we present the results obtained with the proposed method. Moreover, we provide a comparison with the curvature diffusion method [9], which we refer to as “curvature,” and reconstruction with total variation regularization (TV). The TV-based reconstruction was implemented using 30 iterations of gradient descend.

**Figure 5:**
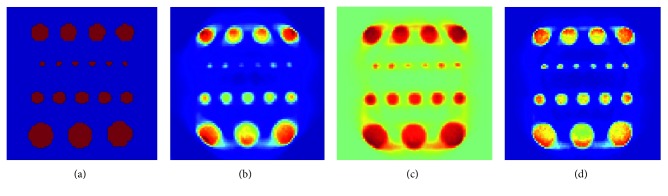
Reconstruction of the phantom with a row of holes: (a) ground truth, (b) curvature method, (c) TV, and (d) proposed method.

The reconstruction of the phantom in Figure 3 is shown in Figure 5 for the different methods. It is evident that all methods provide a good reconstruction for the cylinders of a radius of 4 mm or more. However, the proposed method preserves the rods of 2 mm better; these small cylinders are the most affected by the diffusion procedure of the curvature method and TV method, and this does not occur in the proposed method using weighted curvature.

**Figure 6:**
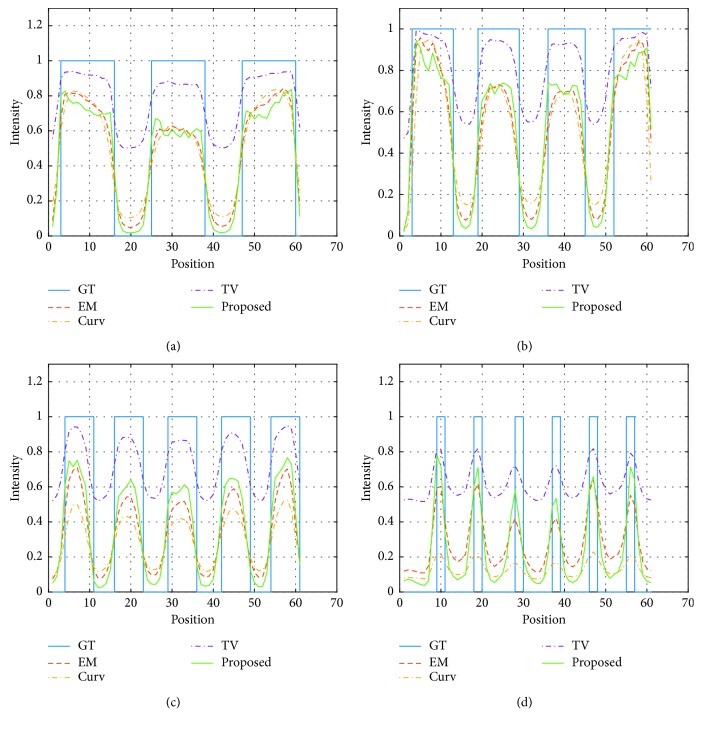
Graphs of the profiles for each cylinder. The solid rectangle shows the ground truth (GT) taken for each method: (a) 5 mm, (b) 4 mm (c) 3 mm, and (d) 2 mm.

We also show graphs of the profiles for each cylinder in Figure 6. The graphs quantitatively confirm the better preservation of the cylinders reconstructed using the proposed method.

Additionally, Table 1 presents measures of PSNR and SSIM between the GT and the methods evaluated. The SSIM measures seem to reflect that all methods do not alter significantly the base structure of the GT image; however, the PSNR indicates that there is less error in the proposed method.

**Figure 7:**
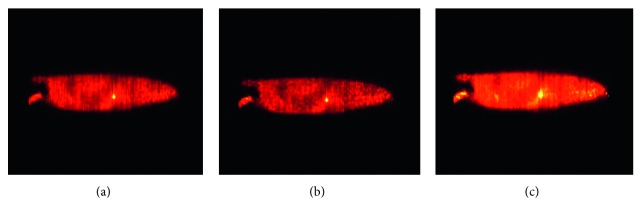
Reconstruction of the Moby phantom: (a) curvature diffusion method, (b) TV, and (c) proposed method.

The reconstructions of the MOBY phantom with lesions are presented in Figure 7. The data obtained were volumetric, but only the slices containing the simulated lesion are shown.

**Figure 8:**
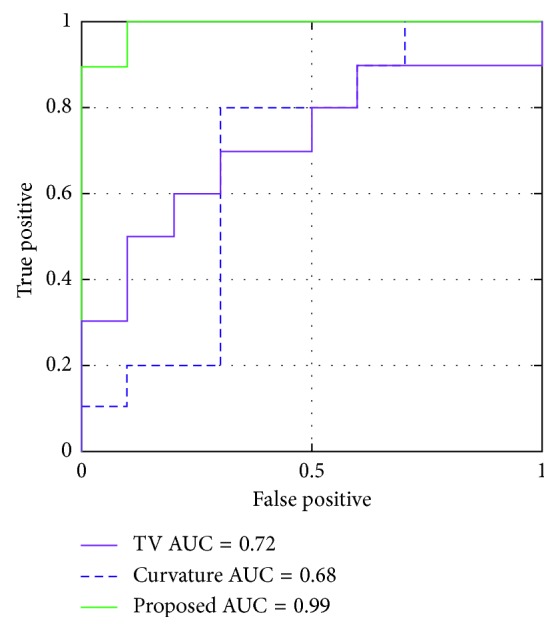
Receiver operating characteristic (ROC) curves obtained from the channelized Hotelling observer, with the signal-known-exactly task. The signal shows a lesion in the liver.

Figure 8 depicts the results of the ROC analysis. The graphs show the calculated ROC for each method and obtained AUC. In this case, the proposed method outperformed the curvature and TV methods, as the proposed method preserved most of the lesion and was most easily detected. This contrasted with the curvature method, which uses a diffusion scheme to regularize the problem.

**Figure 9:**
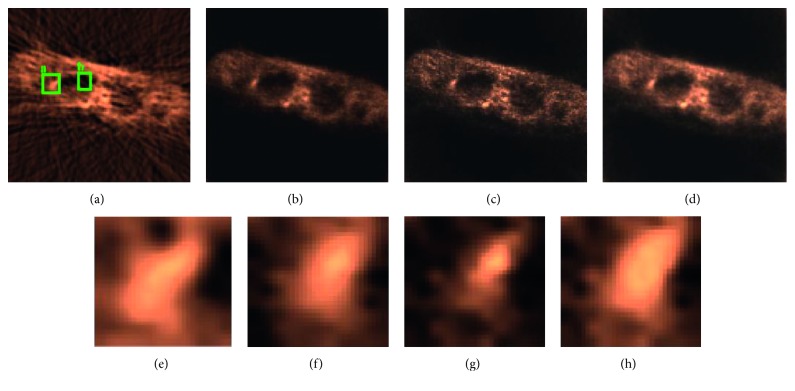
Reconstruction with (a) FBP, (b) curvature diffusion, (c) TV, and (d) proposed method. The square “a” is the zoomed-in region in (e) FBP, (f) curvature diffusion, (g) TV, and (h) the proposed method.

For the study with measured data, we present the results for each reconstruction; these are shown in Figure 9. We also show zoomed-in images of a selected region in Figure 9(e) filtered back projection (FBP), Figure 9(f) for the curvature, Figure 9(g) for TV, and Figure 9(h) for the proposed method. In these images, it is clear that the TV and proposed method provided more definition of the image structure, which appeared more blurred when curvature diffusion was used, while the TV method have good contrast; there exist some artefacts that could be caused by the stair case effect.

In Table 2, it is shown the results of applying the contrast resolution metric [18] to the data using the regions “a” and “b” depicted in Figure 9.

From Table 2, it can be observed that the proposed method achieves better results than the other methods. Although contrast in the TV image is visually good, the amount of noise or artefacts decreases the metric of contrast resolution with respect to the curvature and proposed method. One drawback of the proposed method is that, in some areas, especially those with high counts, the proposed method has more diffusion than TV and curvature methods; these effects can be seen in the lesion of Figure 7 and the lower central region of Figure 9.

## 4. Conclusions

In this paper, we presented a new algorithm for the reconstruction of PET images. The inverse problem was posed as a variational problem, and the use of WGC was proposed. When this term was used as a regularization term, it helped in preserving important structures in the image, such as the borders. It was also capable of preserving structures of low gradient magnitude, such as ramp-like structures, and removing noise present in the image. These are all desirable properties for a reconstruction scheme.

We performed several experiments, which confirmed the above properties when the scheme was applied to lesion detection and contrast preservation. We compared our proposed method with a state-of-the-art method that uses GC without spatial weighting and TV. The obtained results showed that our algorithm performed better than TV and GC without spatial weighting. In future work, we intend to modify the weights of the regularized scheme to provide better adaptation to the PET image.

## Figures and Tables

**Algorithm 1:**
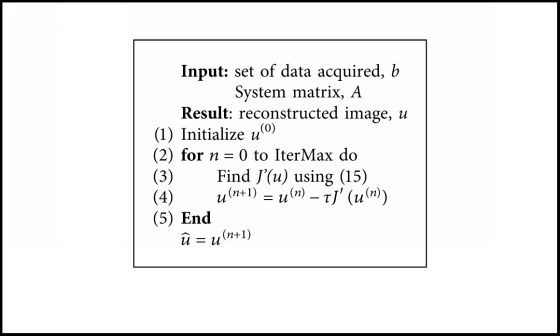
Weighted curvature reconstruction.

**Table 1:** Results of evaluating PSNR and SSIM.

Method	PSNR	SSIM
Curvature	7.89	0.979
TV	6.63	0.972
Proposed	8.25	0.980

**Table 2:** Contrast resolution.

Method	Contrast resolution
Curvature	0.407
TV	0.224
Proposed	0.430

## Data Availability

Phantom data used in the experiments available from https://web.eecs.umich.edu/∼fessler/result/et/ The MOBY is no available, the phantom is Licensed by Segars, William Paul, https://www.ideaconnection.com/patents/6082-4D-Digital-Mouse-Whole-Body-MOBY-Phantom.html.
